# A common functional variant on the pro-inflammatory Interleukin-6 gene may modify the association between long-term PM_10_ exposure and diabetes

**DOI:** 10.1186/s12940-016-0120-5

**Published:** 2016-02-24

**Authors:** Ikenna C. Eze, Medea Imboden, Ashish Kumar, Martin Adam, Arnold von Eckardstein, Daiana Stolz, Margaret W. Gerbase, Nino Künzli, Alexander Turk, Christian Schindler, Florian Kronenberg, Nicole Probst-Hensch

**Affiliations:** Swiss Tropical and Public Health Institute, Basel, Switzerland; University of Basel, Basel, Switzerland; Karolinska Institutet, Stockholm, Sweden; Institute of Clinical Chemistry, University Hospital Zurich, Zurich, Switzerland; Clinic of Pneumology and Respiratory Cell Research, University Hospital Basel, Basel, Switzerland; Faculty of Medicine, University of Geneva, Geneva, Switzerland; Zürcher Höhenklinik Wald, Faltigberg-Wald, Switzerland; Division of Genetic Epidemiology, Department of Medical Genetics, Molecular and Clinical Pharmacology, Medical University of Innsbruck, Innsbruck, Austria; Department of Epidemiology and Public Health, Swiss Tropical and Public Health Institute, Socinstrasse 57, CH-4002 Basel, Switzerland

**Keywords:** Particulate matter, Air pollution, Diabetes mellitus, Interleukin-6 gene, Gene-environment interactions, Single nucleotide polymorphisms, Cross-sectional epidemiology

## Abstract

**Background:**

Air pollutants have been linked to type 2 diabetes (T2D), hypothesized to act through inflammatory pathways and may induce interleukin-6 gene (*IL6*) in the airway epithelium. The cytokine interleukin-6 may impact on glucose homeostasis. Recent meta-analyses showed the common polymorphisms, *IL6* -572G > C and *IL6* -174G > C to be associated with T2D risk. These *IL6* variants also influence circulatory interleukin-6 levels. We hypothesize that these common functional variants may modify the association between air pollutants and T2D.

**Methods:**

We cross-sectionally studied 4410 first follow-up participants of the Swiss Cohort Study on Air Pollution and Lung and Heart Diseases (SAPALDIA), aged 29 to 73 years who had complete data on genotypes, diabetes status and covariates. We defined diabetes as self-reported physician-diagnosed, or use of diabetes medication or non-fasting glucose >11.1 mmol/L or HbA1c > 0.065. Air pollution exposure was 10-year mean particulate matter <10 μm in diameter (PM_10_) assigned to participants’ residences using a combination of dispersion modelling, annual trends at monitoring stations and residential history. We derived interaction terms between PM_10_ and genotypes, and applied mixed logistic models to explore genetic interactions by *IL6* polymorphisms on the odds of diabetes.

**Results:**

There were 252 diabetes cases. Respective minor allele frequencies of *IL6* -572G > C and *IL6* -174G > C were 7 and 39 %. Mean exposure to PM_10_ was 22 μg/m^3^. Both variants were not associated with diabetes in our study. We observed a significant positive association between PM_10_ and diabetes among homozygous carriers of the pro-inflammatory major G-allele of *IL6* -572G > C [Odds ratio: 1.53; 95 % confidence interval (1.22, 1.92); *P*_interaction (additive)_ = 0.003 and *P*_interaction (recessive)_ = 0.006]. Carriers of the major G-allele of *IL6* -174G > C also had significantly increased odds of diabetes, but interactions were statistically non-significant.

**Conclusions:**

Our results on the interaction of PM_10_ with functionally well described polymorphisms in an important pro-inflammatory candidate gene are consistent with the hypothesis that air pollutants impact on T2D through inflammatory pathways. Our findings, if confirmed, are of high public health relevance considering the ubiquity of the major G allele, which puts a substantial proportion of the population at risk for the development of diabetes as a result of long-term exposure to air pollution.

**Electronic supplementary material:**

The online version of this article (doi:10.1186/s12940-016-0120-5) contains supplementary material, which is available to authorized users.

## Background

Evidence suggests a positive association between ambient air pollution and risk of type 2 diabetes (T2D) [1–3]. This association is hypothesized to be mediated through inflammatory mechanisms. Experimental evidence [4, 5] suggests subclinical systemic inflammation occurring at several sites including adipose tissue, liver, skeletal muscles and the autonomic nervous system, with resultant insulin resistance, the hallmark of T2D. On the population level, acute and long-term exposure to ambient air pollution has been linked to raised markers of inflammation including circulating C-reactive proteins (CRP) [6, 7] IL-6, [8] fibrinogen [9, 10] vascular and intracellular adhesion molecules [11]. Indeed, air pollution is thought to accelerate pro-thrombotic state following lung inflammation through an IL-6 dependent pathway [12, 13]. Exposure to industrial particulate matter has been shown to induce IL-6 genes in human airway epithelial cells [14]. Likewise, exposure of mice to particulate matter induced the expression of genes involved in inflammation, lipid metabolism and atherosclerosis [8].

IL-6 in itself may be related to the development of type 2 diabetes [15]. Elevated levels of IL-6 were associated with higher incidence of type 2 diabetes [16, 17], and animal studies also showed IL-6 to inhibit insulin secretion from islet cells following glucose stimulation [18]. Other in vitro studies also showed negative impacts of IL-6 on insulin sensitivity through reduced insulin receptor expression [19] and adiponectin gene expression [20] in adipocytes. Among T2D patients, IL-6 was associated with whole-body insulin resistance and hyperglycemia [21]. Raised IL-6 levels were also associated with hyperinsulinemia in patients without T2D [22].

*IL6* gene plays an important role in the regulation of systemic inflammatory pathways. Some common single nucleotide polymorphisms (SNPs), including *IL6* -572G > C and *IL6* -174G > C have been shown to influence the levels of circulatory IL-6 [23, 24], as well as circulating CRP [25]. In these studies, the major G alleles of both variants were identified as the pro-inflammatory alleles. Recent meta-analyses of 11,681 individuals of Asian and European descent showed the G allele of *IL6* -572G > C to be associated with increased risk of T2D [26] whereas another study of 22,626 individuals of European descent showed the C allele of *IL6* -174G > C to be associated with decreased risk of T2D [27].

Studying gene-environment interactions helps to better understand aetiologic mechanisms and causality of exogenous factors, in this case, air pollution, and to identify population at increased risk of adverse health effects of environmental exposures. We hypothesize, based on the above evidence, that the common functional SNPs, *IL6* -572G > C and *IL6* -174G > C, may modify the existing association between air pollutants and diabetes [28].

## Methods

### Study population

**Fig. 1:**
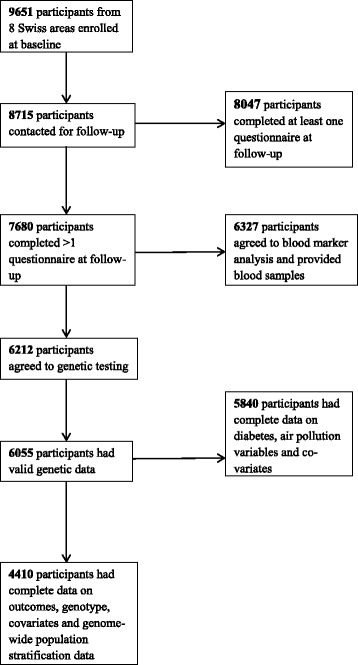
Algorithm for participant inclusion in the present study

We studied participants of the Swiss Cohort Study on Air Pollution and Lung and Heart Diseases in Adults (SAPALDIA). This study has been described elsewhere in detail [29]. Briefly, it consists of a population-based sample of 9651 adults, aged 18–60 when they were recruited at baseline (1990/1991) from eight communities reflecting the diverse geographic and climatic features of Switzerland. Participants underwent health interviews and physical examinations. At first follow-up (2001/2002) which additionally included blood marker and genetic assays, 8047 participants completed at least the screening questionnaire [30]. 6212 participants at first follow-up, consenting to genetic assays were genotyped for *IL6* -572G > C and *IL6* -174G > C in those studies. For the present analysis, we studied 4410 follow-up participants, aged 29–73 years, with complete data on diabetes status, selected covariates, and population stratification data from genome-wide association studies. The algorithm for participant selection is shown on Fig. 1.

Participants provided informed consent for participation in the health interviews, physical examinations, blood marker and genetic assays. Ethical clearance for the SAPALDIA study was obtained from the Swiss Academy of Medical Sciences, the National Ethics Committee for Clinical research (UREK, Project Approval Number 123/00) and the Ethics Committees of the eight participating communities including Basel, Wald, Davos, Lugano, Montana, Payerne, Aarau and Geneva.

### Identification of diabetes cases

Participants were identified as having diabetes if they 1) reported physician diagnosed diabetes or 2) use of diabetes medication in the past month or 3) had non-fasting blood glucose >11.1 mmol/L or 4) HbA1c >0.065. Non-fasting blood glucose was measured in all participants whereas HbA1c was only measured if non-fasting glucose >6.1 mmol/L [28]. The lack of data on diabetes status at baseline precluded the study of incident diabetes. Also, we could not exclude type 1 diabetes (T1D) and have assumed the majority of these cases to be T2D considering that on the average, >90 % of adult diabetes are T2D [31].

### Individual assignment of air pollution exposure

We considered 10-year means of PM_10_ (particulate matter <10um in diameter) as our pollutant exposure measure of interest. In our previous studies on diabetes [2] and metabolic syndrome [32], associations with nitrogen dioxide (NO_2_) disappeared in two-pollutant models that included NO_2_ and PM_10_, whereas those of PM_10_ remained unchanged. Therefore we did not consider NO_2_ in this study. Estimates of PM_10_ exposure were assigned to participants’ residential addresses using dispersion models for the years 1990 and 2000 (respective years before baseline and follow-up) [33]. This model incorporated data from meteorology, topography and several emission inventories including industrial, agricultural, heavy equipment and traffic at a resolution of 200 × 200 m [34]. Annual PM_10_ levels measured at fixed monitoring stations across Switzerland and participants’ residential histories were further used to derive estimates of mean PM_10_ exposure at participants’ residential addresses over the 10-year period preceding the first follow-up health examination [28].

Genotyping of candidate SNPs, *IL6* -572G > C and *IL6* -174G > C.

Genomic DNA was extracted from ethylenediaminetetraacetic acid (EDTA)-buffered whole blood using PUREGENE DNA Purification Kit (GENTRA Systems, Minneapolis, USA) [30]. Genotyping for these polymorphisms was done using 5’-nuclease fluorescent polymerase chain reaction (Taqman) assay (Applera Europe, Rotkreuz, Switzerland). Detection of end-points was done using a 7000 ABI System detection device (ABI, Rotkreuz, Switzerland) [35]. Genotyping call rate was >97.5 %. A random selection of 638 samples (10 %) was genotyped twice for quality control and repeated genotypes were 100 % concordant (R^2^ = 1; *P* < 0.001). Subsamples of 3015 and 1612 SAPALDIA participants had whole genome genotyping using the Illumina HumanOmniExomeExpress BeadChip and Illumina 610 K quad BeadChip (Illumina, San Diego, CA, USA) respectively. In this combined SAPALDIA subsample which are predominantly of Caucasian ancestry, we derived ten population stratification components using multidimensional scaling analysis (Plink v1.07 [36]) on 72,122 SNPs with MAF > 1 % and genotyping call rate >97 %, present on both genotyping arrays.

### Potential confounders

We considered the following characteristics, based on our previous publication on air pollution and diabetes [28], as potential confounders: age (years; continuous), sex, years of formal education (≤9; >9), neighbourhood socio-economic index developed from a principal component analysis including occupation and educational level of household head, median rent and number of persons living in a household, expressed as a percentage [37]. We also considered smoking history (current, former and never smoker; and smoked pack-years computed by multiplying number of cigarette packs per year and number of smoking years), exposure to passive smoke (yes/no), occupational exposure to vapours gases, dusts and fumes (yes/no) daily consumption of at least one portion of fruits and vegetables (never; ≤3 days/week; >3 days/week respectively). We also considered alcohol consumption (including beers, wines, spirits and liquors: never; ≤1glass/day; >1glass/day); hours per week of vigorous physical activity defined as taking part in activities that make one sweat or out of breath (<0.5; ≥0.5), body mass index (BMI; kg/m^2^, continuous) and genome-wide population stratification components.

### Statistical analyses

We assessed linkage disequilibrium between *IL6* -572G > C and *IL6* -174G > C and tested both candidate SNPs for Hardy-Weinberg equilibrium (HWE) among the genotyped participants regardless of inclusion in the study. We computed an *IL6* genetic risk score (IRS) by summing up the risk alleles (number of G alleles coded as 0, 1 and 2) across both SNPs. We summarized the characteristics of 4410 included participants based on their genotype and also by inclusion and exclusion status. We applied a mixed logistic regression model, with a random intercept by study area to explore the associations of diabetes with both SNPs, IRS and with PM_10_. We generated interaction terms between PM_10_ and candidate SNPs (including IRS) and assessed their associations with prevalent diabetes. Interaction analyses with candidate SNPs involved additive, recessive and co-dominant models. Stratifying by genotype, we assessed the associations between PM_10_ and diabetes, to identify genotype-specific associations. We also stratified by groups of IRS indicating increased inflammatory risk. Since this study included 46 % of baseline participants which differed in several sociodemographic characteristics (Additional file 1: Table S1), we assessed the effect of potential participant selection bias on our results using inverse probability weighting by applying the inverse of the probability of participating in present study derived at baseline (using variables that significantly predicted participation in the present study), to the primary model in this study. All analyses were performed using a primary model which included, after stepwise selection, participants’ age, sex, educational attainment, neighbourhood socio-economic index, smoking status, smoked pack-years, exposure to passive smoke, occupational exposure to vapours, gases, dusts and fumes, consumption of fruits and vegetables, vigorous physical activity, BMI and genome-wide population stratification components and applying a random intercept by study area.

We performed several sensitivity analyses. We defined diabetes based on each of the diagnostic criteria (excluding the cases identified only by alternative criteria). We repeated the interaction analyses using mixed logistic regression models with random slopes by study area to explore if study area influenced any of the observed interactions. All statistical analyses were performed with STATA software, version 14 (Stata Corporation, Texas).

## Results

There were 252 diabetes cases in this study. Mean exposure to PM_10_ was 22.6ug/m^3^ and mean IRS was 3 risk alleles. *IL6* -572G > C and *IL6* -174G > C were not in linkage disequilibrium (R^2^ = 0.02; D’ = 1.0). The results of the HWE test for *IL6* -572G > C and *IL6* -174G > C, which have respective minor allele frequency of 7 and 39 % in the SAPALDIA population are shown in Table 1. *IL6* -174G > C was in HWE in both cases and controls whereas *IL6* -572G > C only reached HWE among the diabetes cases (*P* = 0.636) and not among those without diabetes (*P* = 0.006). The overall HWE test for *IL6* -174G > C and *IL6* -572G > C was 0.408 and 0.006 respectively (Table 1). In the European study of ~6000 participants reporting an association between *IL6* -572G > C and T2D, this functional SNP (having MAF = 5 %) was also not in HWE [38]. Since only 30 participants carry the minor CC genotype of *IL6* -572G > C, we present the results for this SNP as GG vs. GC + CC, which yields better statistical power.

**Table 1:** Distribution of *IL6* -572 G > C and *IL6* -174 G > C genotypes and alleles by diabetes status

Genotype	Diabetes	No diabetes
	*N* = 286	*N* = 5554
*IL6* -572 G > C^*^		
Genotype		
GG	248 (86.7)	4896 (88.2)
GC	36 (12.6)	623 (11.2)
CC	2 (0.7)	35 (0.6)
Allele		
G	532 (93)	10415 (93.7)
C	40 (7)	693 (6.3)
*IL6* -174 G > C^**^		
Genotype		
GG	111 (39)	2081 (37)
GC	135 (47)	2614 (47)
CC	40 (14)	865 (16)
Allele		
G	357 (62)	6776 (61)
C	215 (38)	4344 (39)

Data are presented as absolute numbers (N) and relative numbers (%) in parentheses

^*^ *P*-value for Hardy-Weinberg Equilibrium (HWE) test in diabetes cases = 0.585; no diabetes = 0.006; overall = 0.006. *P*-value for Fisher’s exact test = 0.664

^**^ *P*-value for Hardy-Weinberg Equilibrium test in diabetes cases = 0.918; no diabetes = 0.352; overall = 0.408. *P*-value of Chi-square test = 0.762

Compared to the carriers of the major GG genotype, carriers of the GC + CC genotype of *IL6*-572G > C had higher body mass index and PM_10_ exposure whereas carriers of CC genotype of *IL6* -174G > C smoked more, consumed more alcohol and had lower exposure to PM_10_ and there was a significant difference in genotype distribution across areas (Table 2). There was no significant difference in diabetes prevalence across genotypes for both polymorphisms (Table 2). Additional file 1: Table S1 shows the differences in these characteristics between the included and excluded participants. There were significant differences in most of the participants’ characteristics including diabetes prevalence and PM_10_ exposure, but the prevalence of these characteristics were generally higher among the included participants compared to the excluded ones (Additional file 1: Table S1). The *IL6* -572G > C and *IL6* -174G > C genotypes, and IRS were similarly distributed between both groups (Additional file 1: Table S1).

**Table 2:** Characteristics of participants by *IL6* -572 G > C and *IL6* -174 G > C genotypes

	*IL6* -572 G > C			*IL6* -174 G > C			
	GG (*N* = 3891)	GC + CC (*N* = 519)	*P* (Chi^2^)	GG (*N* = 1618)	GC (*N* = 2110)	CC (*N* = 682)	*P* (Chi^2^)
Proportion (%)							
Females	48.4	49.1	0.768	48.3	48.1	50.6	0.509
Education ≥9 years	94.9	93.2	0.107	94.4	94.9	95.0	0.721
Never-smokers	44.3	45.6	0.490	42.4	44.8	48.1	0.036
Passive smoke exposure	46.4	46.6	0.927	48.6	45.4	44.4	0.078
Occupational VGDF exposure	42.8	42.4	0.845	43.9	42.6	40.9	0.398
Alcohol intake ≤1glass/day	91.0	91.4	0.620	89.8	91.2	93.0	0.050
Alcohol intake >1glass/day	9.0	9.6		10.2	8.8	7.0	
Portion of raw vegetables ≤3 days/week	18.2	20.8	0.150	17.4	19.1	19.4	0.351
Portion of raw vegetables >3 days/week	81.8	79.2		82.6	80.9	80.6	
Portion of fruits ≤3 days/week	35.8	35.6	0.923	35.0	36.2	36.7	0.657
Portion of fruits >3 days/week	64.2	64.4		65.0	63.8	63.3	
Portion of citrus fruits ≤3 days/week	64.2	64.0	0.909	64.2	63.9	64.8	0.917
Portion of citrus fruits >3 days/week	35.8	36.0		35.8	36.1	35.2	
Vigorous physical activity <0.5 h/week	35.5	37.2	0.449	37.3	34.6	34.2	0.082
Vigorous physical activity ≥0.5 h/week	64.5	62.8		62.1	65.4	65.8	
Diabetes cases	5.5	6.7	0.260	6.2	5.5	4.8	0.430
Areas: Basel	11.5	12.9	0.062	10.5	11.6	14.8	<0.001
Wald	19.1	19.1		19.0	18.6	20.8	
Davos	7.7	6.9		7.5	7.9	7.0	
Lugano	12.6	14.6		15.0	12.6	8.4	
Montana	11.1	6.9		9.2	11.2	12.0	
Payerne	13.1	12.7		13.3	12.1	15.2	
Aarau	16.6	19.3		16.9	17.6	14.4	
Geneva	8.4	7.5		8.6	8.4	7.3	
Means (SD)			*T*-test				ANOVA
Age (years)	51.8 (11)	51.3 (11)	0.269	51.8 (11)	51.8 (11)	51.5 (11)	0.732
BMI (kg/m^2^)	25.8 (4.3)	26.3 (4.3)	0.025	26.0 (4.3)	25.8 (4.3)	25.9 (4.3)	0.152
Neighborhood SEI	63.7 (10)	63.8 (10)	0.827	63.5 (10)	63.9 (10)	63.8 (9)	0.455
10-year mean PM_10_ (μg/m^3^)	21.8 (7.3)	22.8 (7.2)	0.005	22.3 (7.4)	21.9 (7.3)	21.5 (6.9)	0.030
Pack-years of smoking^a^	0 (14)	0.1 (16)	0.296	0.3 (16)	0 (16)	0 (14)	0.524

VGDF: vapours, gases, dusts and fumes; SD: standard deviation; BMI: body mass index; SEI: socio-economic index; PM_10_: particulate matter <10 μm in diameter. ^a^values represent median (interquartile range) and *P*-values represent significance level of median test

The positive association between air pollutants and diabetes, which we previously observed in our previous study of 6392 participants at this follow-up study [28], persisted in the present sample. The adjusted odds of diabetes increased by 47 % (95 % CI: 1.21, 1.78) per 10ug/m^3^ of exposure to PM_10_. We did not observe any significant association between the candidate *IL6* SNPs and diabetes, across three genetic models. We also did not observe significant associations between IRS and diabetes in our sample (Additional file 1: Table S2).

**Fig. 2:**
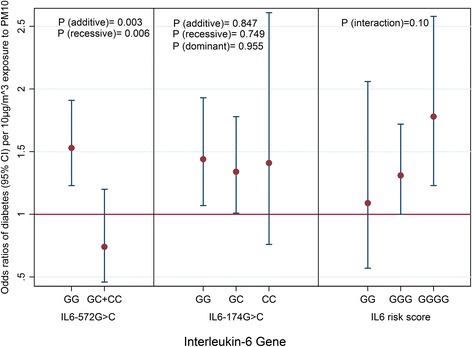
Interaction between PM_10_ and *IL6* polymorphisms on odds of diabetes. Odds ratio values represent percentage increase of odds of diabetes per 10 μg/m^3^ increase in PM_10_ exposure adjusted for potential selection bias. PM_10_: particulate matter <10 μm in diameter. All associations are adjusted for body mass index, age, sex, socio-economic status, smoking habits, consumption of alcohol, fruits and vegetables, physical activity and genome-wide population stratification. Study area was treated as a random effect in all models

Stratified by genotypes, the association between PM_10_ and diabetes was most pronounced among carriers of the major GG pro-inflammatory alleles for both polymorphisms (Fig. 2). We observed significant interactions between PM_10_ and *IL6* -572G > C in the additive and recessive models which became more significant in the models accounting for potential selection bias by IPW and remained significant following Bonferroni correction at *P* = 0.01 (0.05/5) (Table 3). We did not observe any statistically significant interactions with *IL6* -174G > C across genetic models and adjustment for potential selection bias by IPW (Table 3). We observed a positive trend in the association between PM_10_ and diabetes across levels of IRS (Fig. 2). Carriers of four pro-inflammatory G alleles had the highest odds of diabetes per 10ug/m^3^ increase in exposure to PM_10_ (*P*_interaction_ = 0.10) (Fig. 2). Compared to carriers of two pro-inflammatory G alleles, carriers of four risk alleles had 60 % (95 % CI: −15 %, 197 %) higher odds of diabetes per 10ug/m^3^ increase in exposure to PM_10_. These interactions were largely stable to confounder adjustments.

**Table 3:** Associations and interactions between PM_10_ and candidate SNPs on odds of diabetes, applying inverse probability weighting (IPW) to account for potential selection bias

Genotype	Genotype-specific PM_10_ and diabetes association OR (95 % CI)	*P*-value^*^	*P*-value of interaction^**^	*P*-value of interaction^***^	*P*-value of interaction^****^
*IL6* -572G > C					
Adjusted model without IPW					
GG	1.53 (1.22, 1.92)	<0.001	0.031	*n.d.*	0.058
GC + CC	0.87 (0.51, 1.49)	0.618			
Adjusted model applying IPW					
GG	1.53 (1.23, 1.91)	<0.001	0.003	*n.d.*	0.006
GC + CC	0.74 (0.46, 1.20)	0.225			
*IL6* -174G > C					
Adjusted model without IPW					
GG	1.49 (1.09, 2.04)	0.012	0.763	0.966	0.645
GC	1.35 (1.01, 1.80)	0.046			
CC	1.43 (0.80, 2.54)	0.226			
Adjusted model applying IPW					
GG	1.44 (1.07, 1.93)	0.016	0.847	0.955	0.749
GC	1.34 (1.01, 1.78)	0.044			
CC	1.41 (0.76, 2.61)	0.226			

Adjusted models include age, sex, educational attainment, neighborhood-level socio-economic status, smoking status, pack-years of smoking, exposure to passive smoke and occupational dusts and fumes, dietary fibre intake, alcohol consumption, physical activity, body mass index (BMI), PM_10_. Study area was treated as random effects in all models. OR: odds ratio; CI: confidence intervals; OR values represent percent increase in odds of diabetes per 10 μg/m^3^ increase in PM_10_ exposure. PM_10_: particulate matter <10 μm in diameter.^*^ *P*-value of genotype specific association between PM_10_ and diabetes.^**^ additive model (per G allele); ^***^dominant model (GG + GC vs. CC); ^****^recessive model (GG vs. GC + CC); *n.d.*: not done due to the few number of CC allele carriers

Sensitivity analyses showed very similar results. Defining diabetes according to each of the classification criteria showed very similar results with significant interactions in the additive and recessive genetic models (Table 4). Odds of diabetes identified through blood tests among GG genotype carriers remained positive but less significant (Table 4). Applying a logistic regression model with random slopes by study area also did not change estimates of the interaction between PM_10_ and the candidate SNPs on prevalent diabetes, but changed the estimated association between PM_10_ and diabetes among the pro-inflammatory GG genotype carriers (Table 4). In a sub-sample of 2825 non-asthmatic participants who were genotyped using the Illumina Human Omni Exome Express Bead Chip, where *IL6* -572G > C was in HWE (Pearson’s correlation R^2^ between both SNPs =1), associations persisted among pro-inflammatory GG carriers [OR: 1.64 (1.24, 2.16)] and interactions persisted in the models accounting for potential selection bias (*P*_(additive)_ =0.053; *P*_(recessive)=_ 0.048).

**Table 4:** Other Sensitivity Analyses

Sensitivity analysis	Genotype	Adjusted OR (95 % CI)	*P*-value^*^	*P*-value^**^	*P*-value^***^	*P*-value^****^
Diabetes defined as self-reported physician diagnosis and medication use [N(diabetes) = 196]	*IL6* -572G > C					
	GG	1.41 (1.11, 1.79)	0.005	0.001	*n.d.*	0.004
	GC + CC	0.63 (0.38, 1.04)	0.070			
	*IL6* -174G > C					
	GG	1.23 (0.88, 1.71)	0.222	0.931	0.797	0.794
	GC	1.33 (0.98, 1.81)	0.065			
	CC	1.17 (0.58, 2.35)	0.669			
Diabetes defined as self-reported physician diagnosis only [N(diabetes) =193]	*IL6* -572G > C					
	GG	1.43 (1.13, 1.83)	0.003	0.008	*n.d.*	0.003
	GC + CC	0.63 (0.38, 1.04)	0.072			
	*IL6* -174G > C					
	GG	1.23 (0.88, 1.71)	0.226	0.881	0.749	0.694
	GC	1.38 (1.01, 1.88)	0.041			
	CC	1.16 (0.57, 2.33)	0.680			
Diabetes defined as self-reported use of diabetes medication only [N(diabetes) =125]	*IL6* -572G > C					
	GG	1.25 (0.95, 1.66)	0.113	0.008	*n.d.*	0.031
	GC + CC	0.49 (0.22, 1.10)	0.085			
	*IL6* -174G > C					
	GG	1.10 (0.72, 1.66)	0.666	0.954	0.600	0.803
	GC	1.24 (0.86, 1.78)	0.246			
	CC	0.90 (0.36, 2.28)	0.829			
Diabetes cases identified from blood tests only [N (diabetes) = 184]	*IL6* -572G > C					
	GG	1.65 (1.27, 2.13)	<0.001	0.002	*n.d.*	0.006
	GC + CC	0.70 (0.39, 1.25)	0.223			
	*IL6* -174G > C					
	GG	1.46 (1.04, 2.06)	0.030	0.738	0.553	0.962
	GC	1.41 (1.00, 1.99)	0.052			
	CC	1.83 (0.86, 3.90)	0.118			
Model applying random slopes for study areas [N(diabetes) = 252]	*IL6* -572G > C					
	GG	0.92 (0.44, 1.92)	0.819	0.004	*n.d.*	0.008
	GC + CC	0.44 (0.19, 1.05)	0.065			
	*IL6* -174G > C					
	GG	0.86 (0.41, 1.83)	0.704	0.814	0.928	0.687
	GC	0.78 (0.36, 1.70)	0.540			
	CC	0.85 (0.33, 2.20)	0.735			

Adjusted models include age, sex, educational attainment, neighborhood-level socio-economic status, smoking status, pack-years of smoking, exposure to passive smoke and occupational dusts and fumes, dietary fibre intake, alcohol consumption, physical activity, body mass index (BMI), PM_10_. Study area was treated as random effects in all models except the model with random slopes for study area.OR: odds ratio; CI: confidence intervals; OR values represent percent increase in odds of diabetes per 10 μg/m3 increase in PM_10_ exposure. PM_10_: particulate matter <10 μm in diameter; *n.d*: not done due to very low sample size for CCgenotype. ^*^ *P*-value of genotype specific association between air pollutant and diabetes. ^**^additive model (per C allele); ^***^dominant model (GG + GC vs. CC); ^****^recessive model (GC + CC vs.GG)

## Discussion

We found a modifying effect of *IL6* -572G > C polymorphism on the association between air pollutants and diabetes, where carriers of the pro-inflammatory GG genotype were most susceptible. These associations were highly stable to confounder adjustments and remained robust across several sensitivity analyses. The lack of interaction with *IL6* -174G > C is supported by the fact that both SNPs are not in linkage disequilibrium. Combining both SNPs into an IRS showed increased association among participants at high genetic risk of inflammation.

The lack of association between PM_10_ and DM among the GG genotype carriers in the fixed effect model could be attributed to variation of PM_10_ constituents across different areas (Pearson’s R for PM_10_ crustal components across four SAPALDIA areas = 0.34) [39]. Due to the fact that the likelihood-ratio tests for interactions between PM_10_ and study area (also between SNP and study area) were non-significant (*P* > 0.2), and that the goal of SAPALDIA air pollution studies is to capture the between area differences in health effects which cannot be explained by the fixed factors in the models, we performed the main analyses using area as a random covariate. In addition, applying the fixed effects model did not change our estimates of interaction between *IL6* polymorphisms and PM_10_ which is our main interest in this study. The reduced significance of interactions among those reporting the use of diabetes medication is most likely to be due to under-reporting of medication use. The absence of any significant association between the *IL6* polymorphisms and diabetes in our sample may be due to our inability to differentiate T1D from T2D, or the relatively small number of cases compared to other studies [26]. Thus, our observation of a significant interaction is surprising given the limited number of cases in our study.

To our knowledge, this is the first evidence on gene-air pollution interaction in adult diabetes. Until now, gene-air pollution interaction studies focused on respiratory and cardiovascular outcomes, exploring diverse candidate genes or polymorphisms, pollutants and outcomes [40–42]. Many of the interacting genes including *IL6*, regulate systemic oxidative stress and inflammatory pathways [41, 43]. *IL6* is one of the genes involved in systemic inflammation by regulating or inducing the production of inflammatory cytokines such as IL-6 and CRP. In-vitro studies also show that exposure to particulate matter induces *IL6* and *CRP* gene expression in epithelial and macrophage cell lines [14, 44, 45]. In addition, polymorphisms on *IL6* have been shown to interact with acute exposure to carbon monoxide and nitrogen dioxide, in eliciting plasma IL-6 response [43].

Our results support the hypothesis that exposure to air pollution may contribute to diabetes aetiology through inflammatory pathways. Since we have analysed two C/G polymorphisms located in the promoter region of *IL6* in our study, a potential mechanism of action could be related to changes in DNA methylation at these sites, affecting *IL6* gene expression. Air pollution exposure was positively associated with methylation of *IL6* in elderly men [46]. Hypomethylation of *IL6* was associated with raised levels of serum IL-6 in patients with rheumatoid arthritis [47], and with body weight among diabetes patients [48]. In contrast, increased methylation of *IL6* was associated with risk of obesity [49] and body weight among patients without diabetes [48]. Furthermore there is suggestive evidence on a potential link between epigenetic changes at inflammatory genes (including *IL6*) and diabetes [50, 51]. While the evidence supports a role of *IL6* methylation with regard to both, air pollution and diabetes, the relevance of hyper- versus hypomethylation and the association with the *IL6* SNPs studied here needs further clarification.

Our study has major strengths. It provides first evidence to our knowledge on gene-air pollution interactions on diabetes risk. It derives from the large database of the population-based SAPALDIA cohort, with well characterized phenotypes, genotypes and lifestyle characteristics. Our air pollution estimates were assigned to participants’ residences and derived from validated models which have been applied to other studies. [33] By taking into account residential histories of participants, we could compute individual estimates of long-term exposure to air pollution, which is a crucial in understanding disease development and progression attributable to air pollution. We minimized outcome misclassification by identifying undiagnosed diabetes cases through additional blood tests.

Our study also has limitations. It has a cross-sectional design hence we cannot infer causality of observed associations. We tried to improve this by estimating air pollution exposure in the ten years prior to the survey where diabetes was assessed. In addition, an exploratory analysis excluding 17 diabetes cases who reported to have started using diabetes medication before 1991 gave very similar results. We could not differentiate T1D from T2D cases and therefore have misclassified a few cases (on the average <10 % as T2D instead of T1D). [31].

One of our functional SNPs of interest, *IL6* -572G > C, was not in HWE (Table 1). SNPs may deviate from HWE due to genotyping error, population stratification or population selection [52, 53]. Therefore, we assessed quality control by genotyping a subsample of our participants using an alternative genotyping array. There was no indication of genotyping errors since both SNPs had a perfect correlation between the two genotyping rounds. Also, we adjusted for population stratification in our study population using genome-wide principal components. It has been shown that SNPs may still deviate from HWE despite controlling for the afore-mentioned reasons [53]. It is also important to note that *IL6* -572G > C was not in HWE in a study linking it to T2D in Europeans [38] and the minor alleles were similarly distributed between the reference study (MAF = 5 %) and our study (MAF = 7 %). Furthermore, the genotype frequencies were also similarly distributed [Diabetes cases- GG: 90.6 %, GC: 9 %, CC: 0.4 %; No diabetes - GG: 86.7 %, GC: 12.6 %, CC: 0.7 % vs. Table 1). We additionally assessed public databases to identify any potential interference on our PCR probe by nearby SNPs but found no evidence for such. We did not measure plasma IL-6 concentrations in our participants due to lack of funds, precluding our assessment of association between candidate SNPs and serum IL-6 levels, but there was a positive correlation between both SNPs and mean high-sensitivity C-reactive proteins (hs-CRP) measured at this first follow-up (*IL6* -572G > C-CC: 1.28 g/l, GC: 1.45 g/l, GG: 1.58 g/l and *IL6* -174G > C-CC: 1.58 g/l; GC: 1.53 g/l; GG: 1.60 g/l). The larger differences in hs-CRP levels associated with *IL6* -572G > C agrees with its larger interaction effect. Lastly, our study had sample size limitations, especially among the CC genotype carriers of *IL6* -572G > C, limiting the statistical power to detect more associations. Despite this, we made some statistically significant observations.

## Conclusions

Our findings suggest that homozygous carriers of the common pro-inflammatory major ‘G’ allele of *IL6* -572G > C polymorphism may be more susceptible to the diabetogenic effects of particulate matter, supporting the relevance of inflammatory pathways in the relationship between air pollution and diabetes. If confirmed, our results are of high public health relevance considering the ubiquity of the major G alleles, which put a substantial proportion of the population at risk for the development of diabetes as a result of exposure to air pollution. Our results therefore call for replication by other longitudinal population-based studies with adequate air pollution, genotype and diabetes information.

## Additional file

Additional file 1: Table S1. Characteristics of included and excluded participants. **Table S2.** Association between functional *IL6* polymorphisms and diabetes. (DOCX 27 kb)

## Abbreviations

BMI: body mass index
DNA: Deoxyribonucleic acid
EDTA: ethylenediaminetetraacetic acid
HbA1C: Glycosylated haemoglobin
hs-CRP: high-sensitivity C-reactive proteins
HWE: Hardy-Weinberg equilibrium
*IL6*: Interleukin-6 gene
IL-6: Interleukin-6 cytokine
NO_2_: nitrogen dioxide
PM_10_: Particulate matter with diameter less than 10 microns
SAPALDIA: Swiss cohort study on air pollution and lung and heart diseases in adults
SNP: single nucleotide polymorphism
T1D: Type 1 diabetes
T2D: Type 2 diabetes.

## References

[1] Eze IC, Hemkens LG, Bucher HC, Hoffmann B, Schindler C, Kunzli N, Schikowski T, Probst-Hensch NM (2015). Association between Ambient Air Pollution and Diabetes Mellitus in Europe and North America: Systematic Review and Meta-Analysis. Environ Health Perspect.

[2] Eze IC, Schaffner E, Fischer E, Schikowski T, Adam M, Imboden M, Tsai M, Carballo D, von Eckardstein A, Kunzli N (2014). Long-term air pollution exposure and diabetes in a population-based Swiss cohort. Environ Int.

[3] Weinmayr G, Hennig F, Fuks K, Nonnemacher M, Jakobs H, Mohlenkamp S, Erbel R, Jockel KH, Hoffmann B, Moebus S (2015). Long-term exposure to fine particulate matter and incidence of type 2 diabetes mellitus in a cohort study: effects of total and traffic-specific air pollution. Environ Health.

[4] Rajagopalan S, Brook RD (2012). Air pollution and type 2 diabetes: mechanistic insights. Diabetes.

[5] Liu C, Ying Z, Harkema J, Sun Q, Rajagopalan S (2013). Epidemiological and experimental links between air pollution and type 2 diabetes. Toxicol Pathol.

[6] Hennig F, Fuks K, Moebus S, Weinmayr G, Memmesheimer M, Jakobs H, Brocker-Preuss M, Fuhrer-Sakel D, Mohlenkamp S, Erbel R (2014). Association between source-specific particulate matter air pollution and hs-CRP: local traffic and industrial emissions. Environ Health Perspect.

[7] Li Y, Rittenhouse-Olson K, Scheider WL, Mu L (2012). Effect of particulate matter air pollution on C-reactive protein: a review of epidemiologic studies. Rev Environ Health.

[8] Brocato J, Sun H, Shamy M, Kluz T, Alghamdi MA, Khoder MI, Chen LC, Costa M (2014). Particulate matter from Saudi Arabia induces genes involved in inflammation, metabolic syndrome and atherosclerosis. J Toxicol Environ Health.

[9] Hoffmann B, Moebus S, Dragano N, Stang A, Mohlenkamp S, Schmermund A, Memmesheimer M, Brocker-Preuss M, Mann K, Erbel R (2009). Chronic residential exposure to particulate matter air pollution and systemic inflammatory markers. Environ Health Perspect.

[10] Hajat A, Allison M, Diez-Roux AV, Jenny NS, Jorgensen NW, Szpiro AA, Vedal S, Kaufman JD (2015). Long-term Exposure to Air Pollution and Markers of Inflammation, Coagulation, and Endothelial Activation: A Repeat-measures Analysis in the Multi-Ethnic Study of Atherosclerosis (MESA). Epidemiology.

[11] Bind MA, Baccarelli A, Zanobetti A, Tarantini L, Suh H, Vokonas P, Schwartz J (2012). Air pollution and markers of coagulation, inflammation, and endothelial function: associations and epigene-environment interactions in an elderly cohort. Epidemiology.

[12] Budinger GR, McKell JL, Urich D, Foiles N, Weiss I, Chiarella SE, Gonzalez A, Soberanes S, Ghio AJ, Nigdelioglu R (2011). Particulate matter-induced lung inflammation increases systemic levels of PAI-1 and activates coagulation through distinct mechanisms. PLoS One.

[13] Mutlu GM, Green D, Bellmeyer A, Baker CM, Burgess Z, Rajamannan N, Christman JW, Foiles N, Kamp DW, Ghio AJ (2007). Ambient particulate matter accelerates coagulation via an IL-6-dependent pathway. J Clin Invest.

[14] Quay JL, Reed W, Samet J, Devlin RB (1998). Air pollution particles induce IL-6 gene expression in human airway epithelial cells via NF-kappaB activation. Am J Respir Cell Mol Biol.

[15] Kristiansen OP, Mandrup-Poulsen T (2005). Interleukin-6 and diabetes: the good, the bad, or the indifferent?. Diabetes.

[16] Pradhan AD, Manson JE, Rifai N, Buring JE, Ridker PM (2001). C-reactive protein, interleukin 6, and risk of developing type 2 diabetes mellitus. JAMA.

[17] Spranger J, Kroke A, Mohlig M, Hoffmann K, Bergmann MM, Ristow M, Boeing H, Pfeiffer AF (2003). Inflammatory cytokines and the risk to develop type 2 diabetes: results of the prospective population-based European Prospective Investigation into Cancer and Nutrition (EPIC)-Potsdam Study. Diabetes.

[18] Sandler S, Bendtzen K, Eizirik DL, Welsh M (1990). Interleukin-6 affects insulin secretion and glucose metabolism of rat pancreatic islets in vitro. Endocrinology.

[19] Lagathu C, Bastard JP, Auclair M, Maachi M, Capeau J, Caron M (2003). Chronic interleukin-6 (IL-6) treatment increased IL-6 secretion and induced insulin resistance in adipocyte: prevention by rosiglitazone. Biochem Biophys Res Comm.

[20] Fasshauer M, Kralisch S, Klier M, Lossner U, Bluher M, Klein J, Paschke R (2003). Adiponectin gene expression and secretion is inhibited by interleukin-6 in 3 T3-L1 adipocytes. Biochem Biophys Res Comm.

[21] Daniele G, Guardado Mendoza R, Winnier D, Fiorentino TV, Pengou Z, Cornell J, Andreozzi F, Jenkinson C, Cersosimo E, Federici M (2014). The inflammatory status score including IL-6, TNF-alpha, osteopontin, fractalkine, MCP-1 and adiponectin underlies whole-body insulin resistance and hyperglycemia in type 2 diabetes mellitus. Acta Diabetol.

[22] Ruge T, Lockton JA, Renstrom F, Lystig T, Sukonina V, Svensson MK, Eriksson JW (2009). Acute hyperinsulinemia raises plasma interleukin-6 in both nondiabetic and type 2 diabetes mellitus subjects, and this effect is inversely associated with body mass index. Metabolism.

[23] Brull DJ, Montgomery HE, Sanders J, Dhamrait S, Luong L, Rumley A, Lowe GD, Humphries SE (2001). Interleukin-6 gene -174 g > c and -572 g > c promoter polymorphisms are strong predictors of plasma interleukin-6 levels after coronary artery bypass surgery. Arterioscler Thromb Vasc Biol.

[24] Sanderson SC, Kumari M, Brunner EJ, Miller MA, Rumley A, Lowe GD, Marmot MG, Humphries SE (2009). Association between IL6 gene variants -174G > C and -572G > C and serum IL-6 levels: interactions with social position in the Whitehall II cohort. Atherosclerosis.

[25] Sainz J, Perez E, Gomez-Lopera S, Lopez-Fernandez E, Moratalla L, Oyonarte S, Jurado M (2008). Genetic variants of IL6 gene promoter influence on C-reactive protein levels but are not associated with susceptibility to invasive pulmonary aspergillosis in haematological patients. Cytokine.

[26] Yin YW, Sun QQ, Zhang BB, Hu AM, Liu HL, Wang Q, Zeng YH, Xu RJ, Zhang ZD, Zhang ZG (2013). Association between the interleukin-6 gene −572 C/G polymorphism and the risk of type 2 diabetes mellitus: a meta-analysis of 11,681 subjects. Ann Hum Genet.

[27] Huth C, Heid IM, Vollmert C, Gieger C, Grallert H, Wolford JK, Langer B, Thorand B, Klopp N, Hamid YH (2006). IL6 gene promoter polymorphisms and type 2 diabetes: joint analysis of individual participants’ data from 21 studies. Diabetes.

[28] Eze IC, Schaffner E, Zemp E, von Eckardstein A, Turk A, Bettschart R, Schindler C, Probst-Hensch N (2014). Environmental tobacco smoke exposure and diabetes in adult never-smokers. Environ Health.

[29] Martin BW, Ackermann-Liebrich U, Leuenberger P, Kunzli N, Stutz EZ, Keller R, Zellweger JP, Wuthrich B, Monn C, Blaser K (1997). SAPALDIA: methods and participation in the cross-sectional part of the Swiss Study on Air Pollution and Lung Diseases in Adults. Soz Praventivmed.

[30] Ackermann-Liebrich U, Kuna-Dibbert B, Probst-Hensch NM, Schindler C, Felber Dietrich D, Stutz EZ, Bayer-Oglesby L, Baum F, Brandli O, Brutsche M (2005). Follow-up of the Swiss Cohort Study on Air Pollution and Lung Diseases in Adults (SAPALDIA 2) 1991–2003: methods and characterization of participants. Sozi Praventivmed.

[31] Alberti KG, Zimmet PZ (1998). Definition, diagnosis and classification of diabetes mellitus and its complications. Part 1: diagnosis and classification of diabetes mellitus provisional report of a WHO consultation. Diabet Med.

[32] Eze IC, Schaffner E, Foraster M, Imboden M, von Eckardstein A, Gerbase MW, Rothe T, Rochat T, Kunzli N, Schindler C (2015). Long-Term Exposure to Ambient Air Pollution and Metabolic Syndrome in Adults. PLoS One.

[33] Liu LJ, Curjuric I, Keidel D, Heldstab J, Kunzli N, Bayer-Oglesby L, Ackermann-Liebrich U, Schindler C (2007). Characterization of source-specific air pollution exposure for a large population-based Swiss cohort (SAPALDIA). Environ Health Perspect.

[34] Swiss Agency for the Environment Forests and Landscape. Modelling of PM10 and PM2.5 ambient concentrations in Switzerland 2000 and 2010. Environ Doc 2003;(Nr. 169).

[35] Imboden M, Nieters A, Bircher AJ, Brutsche M, Becker N, Wjst M, Ackermann-Liebrich U, Berger W, Probst-Hensch NM (2006). Cytokine gene polymorphisms and atopic disease in two European cohorts. (ECRHS-Basel and SAPALDIA). Clin Mol Allerg.

[36] Purcell S, Neale B, Todd-Brown K, Thomas L, Ferreira MA, Bender D, Maller J, Sklar P, de Bakker PI, Daly MJ (2007). PLINK: a tool set for whole-genome association and population-based linkage analyses. Am J Hum Genet.

[37] Panczak R, Galobardes B, Voorpostel M, Spoerri A, Zwahlen M, Egger M (2012). A Swiss neighbourhood index of socioeconomic position: development and association with mortality. J Epidemiol Community Health.

[38] Hamid YH, Rose CS, Urhammer SA, Glumer C, Nolsoe R, Kristiansen OP, Mandrup-Poulsen T, Borch-Johnsen K, Jorgensen T, Hansen T (2005). Variations of the interleukin-6 promoter are associated with features of the metabolic syndrome in Caucasian Danes. Diabetologia.

[39] Aguilera I, Eeftens M, Meier R, Ducret-Stich RE, Schindler C, Ineichen A, Phuleria HC, Probst-Hensch N, Tsai MY, Kunzli N (2015). Land use regression models for crustal and traffic-related PM2.5 constituents in four areas of the SAPALDIA study. Environ Res.

[40] Zanobetti A, Baccarelli A, Schwartz J (2011). Gene-air pollution interaction and cardiovascular disease: a review. Prog Cardiovasc Dis.

[41] Adam M, Imboden M, Boes E, Schaffner E, Kunzli N, Phuleria HC, Kronenberg F, Gaspoz JM, Carballo D, Probst-Hensch N (2014). Modifying effect of a common polymorphism in the interleukin-6 promoter on the relationship between long-term exposure to traffic-related particulate matter and heart rate variability. PLoS One.

[42] Minelli C, Wei I, Sagoo G, Jarvis D, Shaheen S, Burney P (2011). Interactive effects of antioxidant genes and air pollution on respiratory function and airway disease: a HuGE review. Am J Epidemiol.

[43] Ljungman P, Bellander T, Schneider A, Breitner S, Forastiere F, Hampel R, Illig T, Jacquemin B, Katsouyanni K, von Klot S (2009). Modification of the interleukin-6 response to air pollution by interleukin-6 and fibrinogen polymorphisms. Environ Health Perspect.

[44] Vogel CF, Sciullo E, Wong P, Kuzmicky P, Kado N, Matsumura F (2005). Induction of proinflammatory cytokines and C-reactive protein in human macrophage cell line U937 exposed to air pollution particulates. Environ Health Perspect.

[45] Watterson TL, Sorensen J, Martin R, Coulombe RA (2007). Effects of PM2.5 collected from Cache Valley Utah on genes associated with the inflammatory response in human lung cells. J Toxicol Environ Health.

[46] Bind MA, Lepeule J, Zanobetti A, Gasparrini A, Baccarelli A, Coull BA, Tarantini L, Vokonas PS, Koutrakis P, Schwartz J (2014). Air pollution and gene-specific methylation in the Normative Aging Study: association, effect modification, and mediation analysis. Epigenetics.

[47] Ishida K, Kobayashi T, Ito S, Komatsu Y, Yokoyama T, Okada M, Abe A, Murasawa A, Yoshie H (2012). Interleukin-6 gene promoter methylation in rheumatoid arthritis and chronic periodontitis. J Periodontol.

[48] Aumueller E, Remely M, Baeck H, Hippe B, Brath H, Haslberger AG (2015). Interleukin-6 CpG Methylation and Body Weight Correlate Differently in Type 2 Diabetes Patients Compared to Obese and Lean Controls. J Nutrigenet Nutrigenomics.

[49] Na YK, Hong HS, Lee WK, Kim YH, Kim DS (2015). Increased methylation of interleukin 6 gene is associated with obesity in Korean women. Mol Cells.

[50] Gilbert ER, Liu D (2012). Epigenetics: the missing link to understanding beta-cell dysfunction in the pathogenesis of type 2 diabetes. Epigenetics.

[51] Ling C, Groop L (2009). Epigenetics: a molecular link between environmental factors and type 2 diabetes. Diabetes.

[52] Ziegler A, Van Steen K, Wellek S (2011). Investigating Hardy-Weinberg equilibrium in case–control or cohort studies or meta-analysis. Breast Cancer Res Treat.

[53] Hosking L, Lumsden S, Lewis K, Yeo A, McCarthy L, Bansal A, Riley J, Purvis I, Xu CF (2004). Detection of genotyping errors by Hardy-Weinberg equilibrium testing. Eur J Hum Genet.

