# Supplementary material for: A common functional variant on the pro-inflammatory Interleukin-6 gene may modify the association between long-term PM10 exposure and diabetes
Source: Environ Health. 2016 Feb 24;15:39. doi: 10.1186/s12940-016-0120-5 (PMC4765217; doi:10.1186/s12940-016-0120-5)
Supplement: Additional file 1: Table S1. — Characteristics of included and excluded participants. Table S2. Association between functional IL6 polymorphisms and diabetes. (DOCX 27 kb) [file 12940_2016_120_MOESM1_ESM.docx]

**ADDITIONAL FILE**

**A common functional variant on the pro-inflammatory Interleukin-6 gene may modify the association between long-term PM_10_ exposure and diabetes**

Ikenna C. Eze,^1,2^ Medea Imboden,^1,2^ Ashish Kumar,^1,2,3^ Martin Adam,^1,2^ Arnold von Eckardstein,^4^ Daiana Stolz,^5^ Margaret W. Gerbase,^6^ Nino Künzli,^1,2^ Alexander Turk,^7^ Christian Schindler ^1,2^, Florian Kronenberg^8^ and Nicole Probst-Hensch ^1,2^.

^1^Swiss Tropical and Public Health Institute, Basel, Switzerland

^2^University of Basel, Basel, Switzerland

^3^ Karolinska Institutet, Stockholm, Sweden

^4^Institute of Clinical Chemistry, University Hospital Zurich, Switzerland

^5^Clinic of Pneumology and Respiratory Cell Research, University Hospital, Basel, Switzerland

^6^Faculty of Medicine, University of Geneva, Geneva, Switzerland

^7^ Zürcher Höhenklinik Wald, Faltigberg-Wald, Switzerland

^8^Division of Genetic Epidemiology, Department of Medical Genetics, Molecular and Clinical Pharmacology, Medical University of Innsbruck, Innsbruck, Austria

Table of Contents

Table A1. Characteristics of included and excluded participants..............................................2

Table A2. Association between functional *IL6* polymorphisms and diabetes ………………..3

Table A1: Characteristics of included and excluded participants

| Proportion (%) | Included (N=4410) | Excluded % (N) | P-value (Chi^2^) |
| --- | --- | --- | --- |
| Females | 48.5 | 57.6 (5241) | <0.001 |
| Education ≥9 years | 95.0 | 91.0 (5241) | <0.001 |
| Never-smokers | 44.4 | 41.4 (5241) | 0.026 |
| Passive smoke exposure | 46.5 | 47.6 (5241) | 0.414 |
| Occupational VGDF exposure | 42.8 | 41.0 (5241) | 0.180 |
| Alcohol intake ≤1glass/day | 90.0 | 90.9 (2976) | 0.908 |
| Alcohol intake >1glass/day | 9.0 | 9.1 (294) |  |
| Portion of raw vegetables ≤3 days/week | 18.5 | 21.7 (752) | 0.003 |
| Portion of raw vegetables >3 days/week | 81.5 | 78.3 (2518) |  |
| Portion of fruits ≤3 days/week | 35.8 | 33.0 (1112) | 0.030 |
| Portion of fruits >3 days/week | 64.2 | 67.0 (2158) |  |
| Portion of citrus fruits ≤3 days/week | 64.1 | 65.4 (2142) |  |
| Portion of citrus fruits >3 days/week | 35.9 | 34.6 (1128) | 0.320 |
| Vigorous physical activity <0.5 hour/week | 35.7 | 45.5 (1488) |  |
| Vigorous physical activity ≥0.5 hour/week | 64.3 | 54.5 (1782) | <0.001 |
| Diabetes cases | 5.7 | 3.2 (75) | <0.001 |
| *IL6*-572 G>C: GG | 88.2 | 88.0 (1494) | 0.417 |
| GC | 11.1 | 11.6 (197) |  |
| CC | 0.7 | 0.4 (7) |  |
| *IL6*-174 G>C: GG | 36.7 | 38.9 (690) | 0.219 |
| GC | 47.8 | 45.4 (808) |  |
| CC | 15.5 | 15.8 (281) |  |
| Areas: Basel | 11.8 | 15.4 (970) | <0.001 |
| Wald | 19.0 | 15.9 (676) |  |
| Davos | 7.6 | 8.9 (408) |  |
| Lugano | 12.8 | 18.3 (743) |  |
| Montana | 10.6 | 6.2 (326) |  |
| Payerne | 13.0 | 15.7 (919) |  |
| Aarau | 16.9 | 11.2 (551) |  |
| Geneva | 8.3 | 8.4 (632) |  |
| Means (SD) [N] |  |  | P-value (T-test) |
| Age (years) | 51.8 (11.1) | 53.1 (12.0) [5241] | <0.001 |
| BMI (kg/m^2^) | 25.9 (4.3) | 25.9 (4.7) [3270] | 0.874 |
| Neighborhood SEI | 63.7 (9.9) | 62.9 (10.6) [3270] | 0.005 |
| 10-year mean PM_10_ (µg/m^3^) | 22.0 (7.2) | 23.2 (7.2) [5241] | <0.001 |
| *IL6* risk score | 3.1 (0.7) | 3.1 (0.7) [1698] | 0.222 |
| Pack-years of smoking ^a^ | 10.4 (18.0) | 11.8 (19.1) [5241] | 0.069 |

VGDF: vapours, gases, dusts and fumes; SD: standard deviation; BMI: body mass index; SEI: socio-economic index; PM_10_: particulate matter <10μm in diameter. ^a^ values represent median (interquartile range) and P-values represent significance level of median test.

Table A2: Association between functional *IL6* polymorphisms and diabetes

| Model | Reference genotype |  | Crude  OR (95% CI) | Adjusted model  OR (95% CI) | Adjusted model+PM_10_  OR (95% CI) |
| --- | --- | --- | --- | --- | --- |
| *IL6*-572G>C |  |  |  |  |  |
| Additive | Per G allele | Per G allele | 0.91 (0.66,1.26) | 0.94 (0.64,1.39) | 0.95 (0.64,1.40) |
| Co-  dominant | CC | GC | 0.99 (0.23,4.29) | 1.23 (0.20,7.72) | 1.23 (0.20,7.23) |
|  | CC | GG | 0.89 (0.21,3.73) | 1.13 (0.18,7.00) | 1.14 (0.19,7.01) |
| Dominant | CC | GG+GC vs. CC | 0.90 (0.21,3.78) | 1.16 (0.19,7.15) | 1.17 (0.19,7.18) |
| Recessive | GC+CC | GG vs. GC+CC | 0.90 (0.63,1.28) | 0.93 (0.61,1.40) | 0.93 (0.62,1.41) |
| *IL6*-174G>C |  |  |  |  |  |
| Additive | Per G allele | Per G allele | 1.04 (0.87,1.24) | 1.07 (0.87,1.31) | 1.07 (0.87,1.31) |
| Co-  dominant | CC | GC | 1.09 (0.76,1.56) | 1.20 (0.78,1.86) | 1.22 (0.79,1.88) |
|  | CC | GG | 1.10 (0.76,1.59) | 1.19 (0.76,1.86) | 1.21 (0.77,1.88) |
| Dominant | CC | GG+GC vs. CC | 1.09 (0.77,1.54) | 1.20 (0.79,1.81) | 1.21 (0.81,1.83) |
| Recessive | GC+CC | GG vs. GC+CC | 1.03 (0.81,1.32) | 1.03 (0.77,1.38) | 1.03 (0.78,1.38) |
| *IL6* genetic risk score |  |  |  |  |  |
|  | Per G allele | Per G allele | 1.01 (0.86,1.20) | 1.05 (0.86,1.27) | 1.05 (0.86,1.28) |
|  | 2 G alleles | 3 G alleles | 1.12 (0.81,1.54) | 1.14 (0.78,1.66) | 1.16 (0.80,1.68) |
|  | 2 G alleles | 4 G alleles | 1.05 (0.74,1.48) | 1.11 (0.74,1.67) | 1.13 (0.75,1.69) |

Adjusted models include age, sex, educational attainment, neighborhood-level socio-economic status, smoking status, pack-years of smoking, exposure to passive smoke and occupational dusts and fumes, dietary fibre intake, alcohol consumption, physical activity, body mass index (BMI), PM_10_. Study area was treated as random effects in all models. OR: odds ratio; CI: confidence intervals; OR values represent percent increase in odds of diabetes across different genetic models. PM_10_: particulate matter <10μm in diameter.
